# Differential Regulation of Eicosanoid and Endocannabinoid Production by Inflammatory Mediators in Human Choriodecidua

**DOI:** 10.1371/journal.pone.0148306

**Published:** 2016-02-03

**Authors:** M. D. Mitchell, G. E. Rice, K. Vaswani, D. Kvaskoff, H. N. Peiris

**Affiliations:** University of Queensland Centre for Clinical Research, Centre for Clinical Diagnostics, University of Queensland, Royal Brisbane and Women’s Hospital, Queensland, Brisbane, Australia; University of Southampton School of Medicine, UNITED KINGDOM

## Abstract

An increase in intrauterine prostaglandin production is critical for the onset and progression of labor in women and indeed all mammalian species studied. Endocannabinoids can act as substrates for enzymes of the prostaglandin biosynthetic pathways and can be utilized to generate other related compounds such as prostamides. The end products are indistinguishable by radioimmunoassay. We have separated such compounds by mass spectrometry. We now show that inflammatory stimuli such as LPS and proinflammatory cytokines act differentially on these pathways in human choriodecidua and preferentially create drive through to prostaglandin end products. These findings create doubt about the interpretation of data on prostaglandin biosynthesis in intrauterine tissues from pregnant women especially in the presence of an infection. The possibility is raised that separation of these products might reduce variability in results and lead to potential uses for their measurement in the diagnosis of preterm labor.

## Introduction

Preterm delivery (i.e., births occurring <37 weeks gestation) is a major obstetric health problem. In the United States, the preterm birth rate was 11.39% in 2013 [1]. Worldwide, approximately 13 million babies are born prematurely each year [2]. These statistics have remained constant or worsened for decades despite advances in knowledge and medical care. Prematurity is the single most severe complication of pregnancy contributing to poor neonatal outcome. It is strongly associated with low birthweight, increased incidence of perinatal mortality, and greater susceptibility to adult-onset diseases [3]. Parturition is activated by a combination of endocrine and mechanical stimuli from both mother and infant that results in four distinct physiological events: cervical remodeling, uterine contraction, cervical dilatation, and fetal membrane rupture. These events are coordinated by multiple effector pathways, such as NFκB for the activation of inflammation and endocannabinoids and prostaglandin endoperoxidase synthase (PGHS)-2 for uterine contractions. Premature birth may occur when these signaling pathways are blocked, mimicked or subverted such that effector pathways are activated irrespective of fetal development. One such pathway that is coming to prominence is the endocannabinoid regulation of labor. Nevertheless, our understanding of how labor is initiated remains poor.

It is, therefore, vital to obtain a better understanding of the basic mechanisms of preterm birth, because even if existing preventive interventions were fully scaled, fewer than 20% of preterm births would be prevented [4]. Classic studies from Mont Liggins provide what is still today the basic outline of the mechanisms of parturition [5]. Unfortunately, these studies in sheep have not been directly transferable to humans without significant anomalies, as the traditional theory of activation of the fetal hypothalamic-pituitary-adrenal axis and the coordinated stimulation of intrauterine prostaglandin production is not fully consistent with what is known about labor onset in women [6]. What is now unequivocal is that the parturient process in all mammals requires increased intrauterine prostaglandin production which is achieved by arachidonic acid metabolism via fatty acid cyclooxygenase (COX). Treatment with prostaglandins induces labor, and inhibition of prostaglandin biosynthesis prevents labor and delivery [7]. This increase in prostaglandin levels with term labor is a cause and not a result of labor [8] and occurs before the onset of labor [9]. We also know that intrauterine-associated infection is a major cause of premature delivery [10]. Indeed, preterm labor may be associated with an exaggerated production of cytokines [11], which stimulate prostaglandin production from intrauterine tissues [10]. Thus, a better understanding of the mechanisms by which cytokines stimulate prostaglandin biosynthesis could lead to the development of novel approaches for preventing and treating preterm labor.

**Fig 1:**
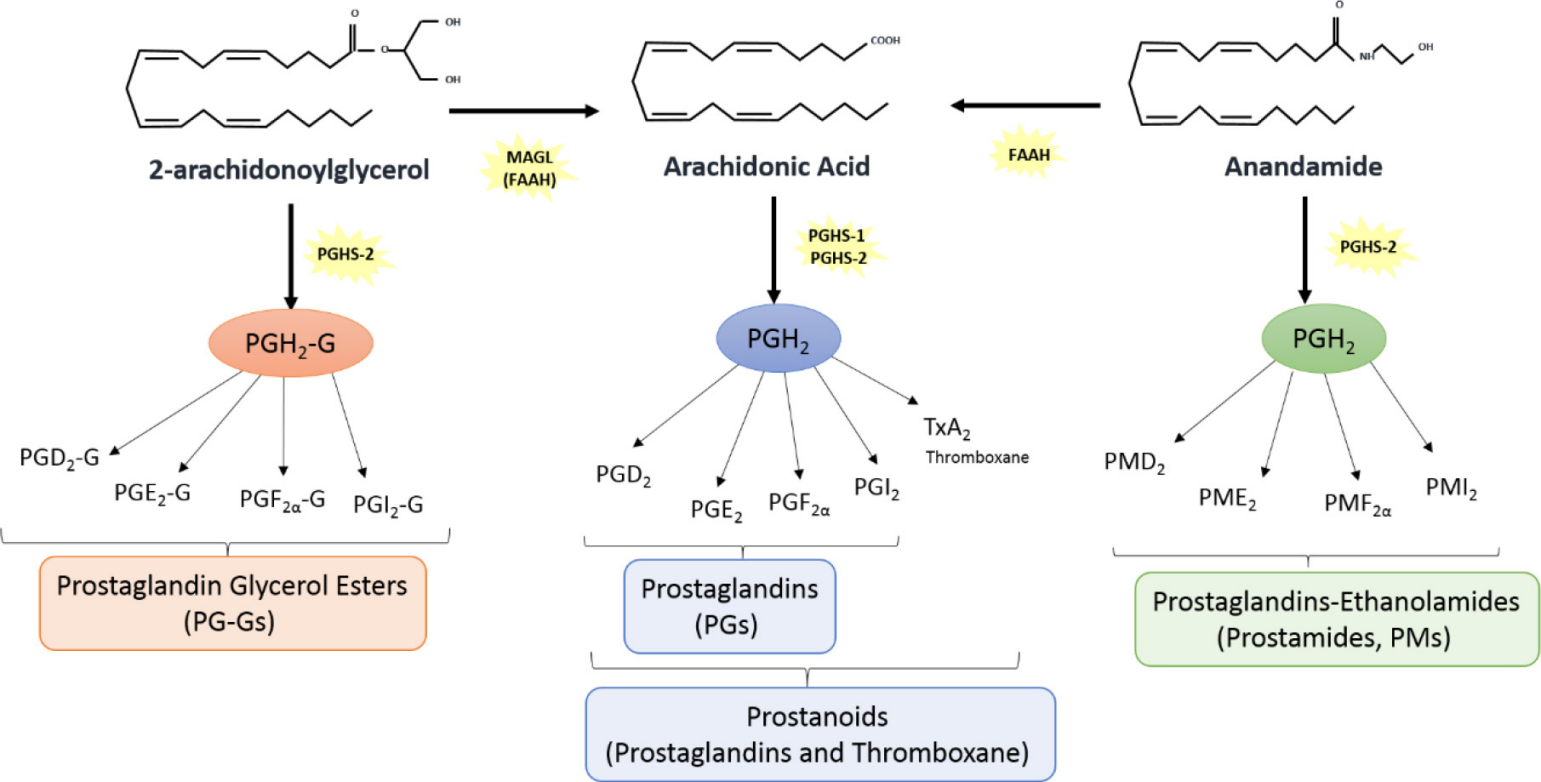
Endocannabinoid pathway. Endocannabinoid pathway giving rise to prostaglandins glycerol esters, prostaglandins and prostamides.

We have shown that anandamide acts as substrate for prostamide production and that stimulation with cytokines mainly induces prostamide output rather than prostaglandin output [12]. Because all presently available antisera to prostaglandins e.g. prostaglandin E_2_ (PGE_2_) recognize prostamides, when a substrate is targeted to the site of inflammation, a prostamide may be secreted rather than a prostaglandin (Fig 1). Hence, the overall response to treatment depends on the properties of the prostaglandin or prostamide formed; these substances have widely divergent contractile activities. Furthermore, this induces variation across studies and affects the reproducibility of diagnostic results. In particular, studies of cytokine effects on prostaglandin biosynthesis, which is a critical step in intrauterine infection-induced preterm labor, may have dramatically different results and may fail to reveal key information about specific cytokines. We have now used the gold standard method of mass spectrometry to identify unequivocally products of endocannabinoid and eicosanoid biosynthetic pathways that are formed upon exposure to inflammatory stimuli of human choriodecidua.

## Materials and Methods

### Patients

Placentae were obtained from women undergoing elective at Caesarean section at term before the onset of labor because of prior Caesarean section or cephalo-pelvic disproportion. Collection was approved by the Human Research Ethics Committees of the Royal Brisbane and Women’s Hospital, and the University of Queensland. Women gave written informed consent for use of placental tissue for research purposes.

### Explant Cultures

Five individual placentae from singleton pregnancies of healthy non-smoking mothers were used in the study. In triplicate, chorio-decidual tissue explants were generated and cultured in 12-well plates as described previously [12, 13]. Briefly, explants were initially placed in DMEM/F12 media supplemented with glutamax (Life Technologies Australia Pty Ltd), 10% FBS (Life Technologies Australia Pty Ltd), and 1% Antibiotic-antimycotic (Life Technologies Australia Pty Ltd) and incubated at 37◦C in humid 5% CO2/95% air for 24 h. After a further 24 h incubation with FBS-free media, explants were treated for a further 24 h with 0, 0.1, 1, or 10 μg/mL lipopolysaccharide derived from Escherichia coli (LPS) (Sigma-Aldrich, Australia); 0, 0.1, 1, or 10 ng/mL interleukin 1beta (IL-1β) (BD Biosciences); or 0, 1, 10, or 100 ng/mL tumor necrosis factor alpha (TNF-α) (BD Biosciences). We have previously shown [12, 13] that these timeframes and condition do not alter explant viability.

### Lipid Analyses

Lipid metabolites were isolated from cell culture media (50 μL) and tissue (30 mg) by extraction in 1% formic acid in cold methanol (Sigma-Aldrich, Australia; 200 μL, containing 250 fmol internal standards) followed by sonication, shaking, and centrifugation (4,500 x g at 4°C) to precipitate proteins. Solid phase clean-up on reverse-phase cartridges (Phenomenex Inc. NSW Australia), using 10% methanol (Sigma-Aldrich, Australia) as a washing solution, removed most water-soluble interferences (e.g., salts from culture media and proteins). Deuterated internal standards (purchased from Cayman Chemicals-Sapphire Bioscience Pty. Ltd) were used to normalize signal response across samples and to account for any differences in extraction recovery (extraction recovery for all eicosanoids was >85%).

Authentic standards were purchased from Cayman Chemicals-Sapphire Bioscience Pty. Ltd Prostaglandin E_2_ Ethanolamide (PGE2-EA), Deuterated Prostaglandin E_2_ Ethanolamide (PGE2-EA-d4), Prostaglandin F_2α_ Ethanolamide (PGF2α-EA), Deuterated Prostaglandin F_2α_ Ethanolamide (PGF2α-EA-d4), Prostaglandin E_2_ (PGE2), Deuterated Prostaglandin E_2_ (PGE2-d4), PGF2α (#10007221), Deuterated Prostaglandin F_2α_ (PGF2α-d4), 13,14-dihydro-15-keto PGF2α (PGFM), Deuterated 13,14-dihydro-15-keto PGF2α (PGFM-d4), Arachidonoyl ethanolamide (Anandamide; AEA), Deuterated Arachidonoyl ethanolamide (AEA-d4), 2-Arachidonoyl glycerol (2-AG), Deuterated 2-Arachidonoyl glycerol (2-AG-d8).

#### Statistical analyses

Statistical differences were determined using the Wilcoxon signed-rank test.

## Results and Discussion

### Results

**Fig 2:**
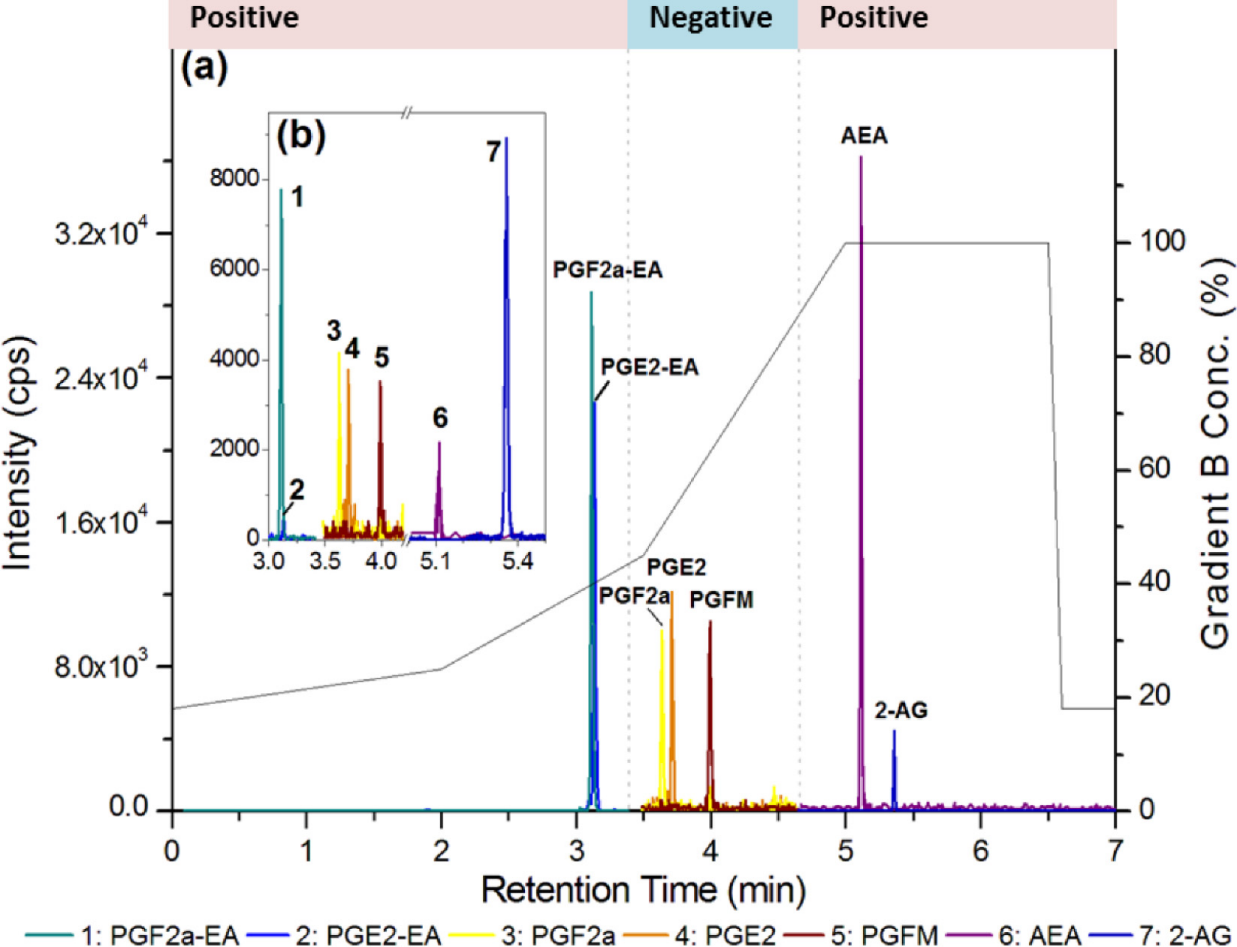
Chromatogram for analysis of prostaglandins, endocannabinoids, and prostamides by LC-MS/MS. Representative chromatogram with gradient conditions, showing polarity switching periods for the concomitant analysis of prostaglandins, endocannabinoids, and prostamides by LC-MS/MS in **(a)** 100 pmol/L standard and **(b)** 10 μL extract of placental choriodecidua.

**Fig 3:**
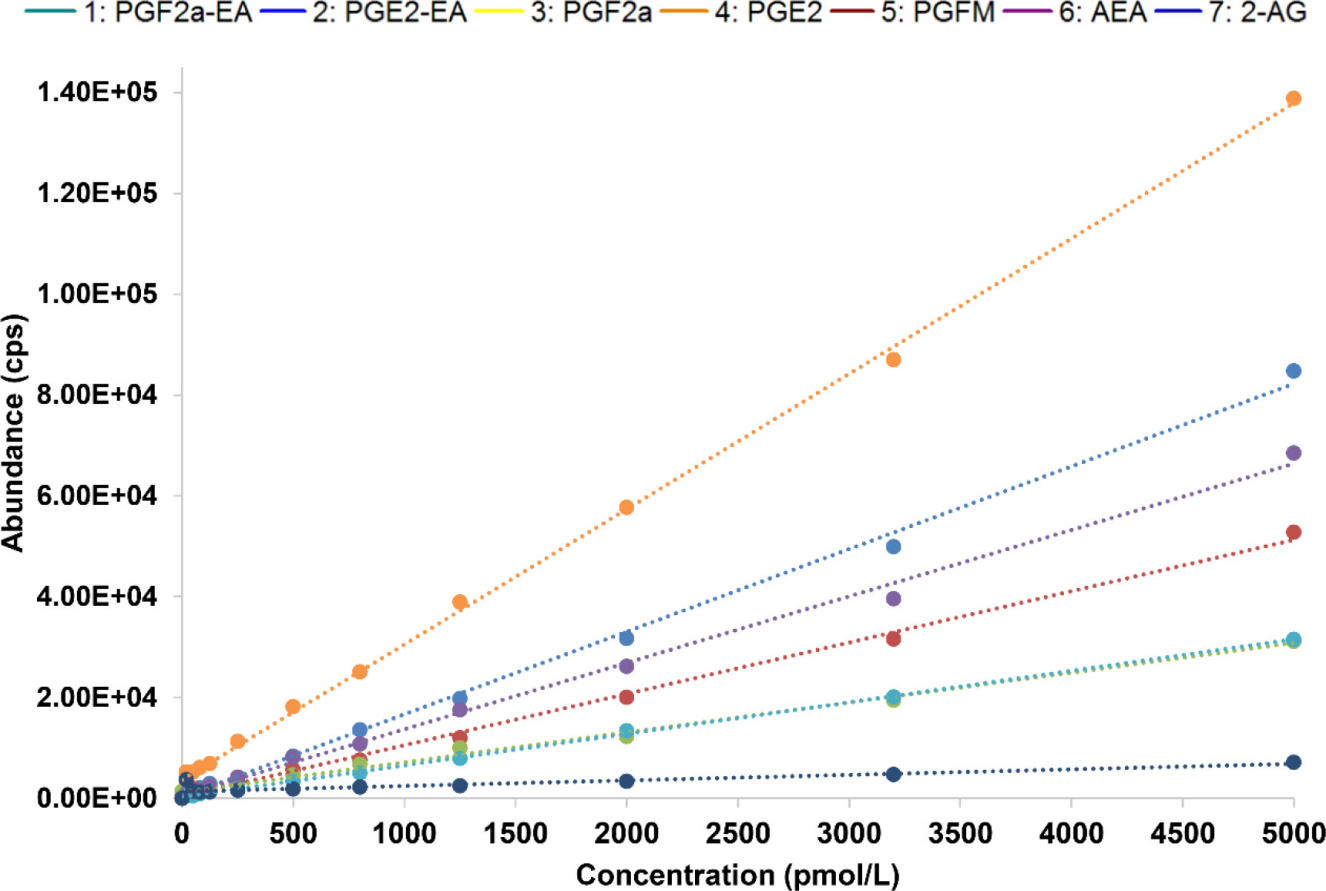
Standard Curve. Linear calibration range of prostaglandins, endocannabinoids, and prostamides (0.01–5.0 nmol/L).

**Fig 4:**
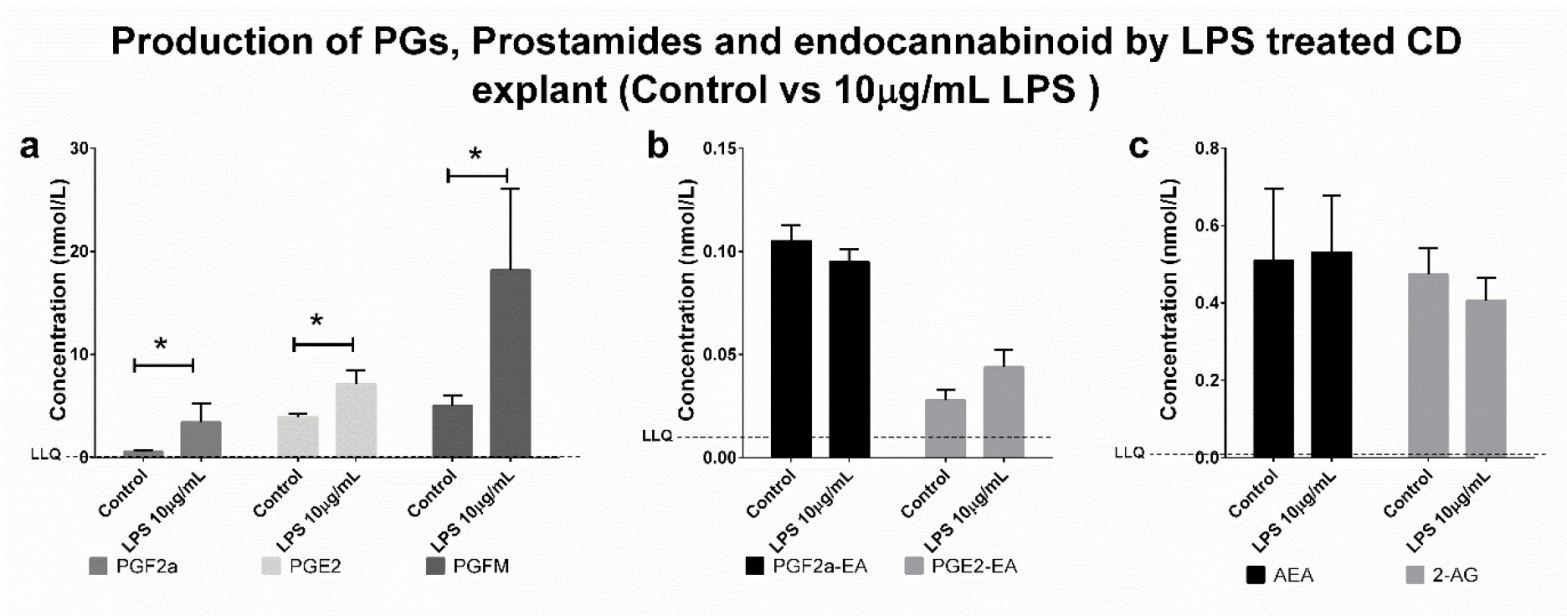
Production of PGs, Prostamides and Endocannabinoids by LPS treated CD explants (control vs 10μg/mL LPS). LC-MS/MS detection of (a) prostaglandins (PGF_2α_, PGE_2_ and PGFM), (b) prostamides (PGF_2α_-EA and PGE_2_-EA) and (c) endocannabinoids (AEA and 2-AG) production by choriodecidual explants treated with 10μg/mL LPS. Dashed line represents the lower limit of quantitation (LLQ). Significant differences in production of the compounds were determined by Wilcoxon signed-rank test. and significance was defined as P<0.05 (* on graphs). Data were presented as the mean ± S.E.M.

**Fig 5:**
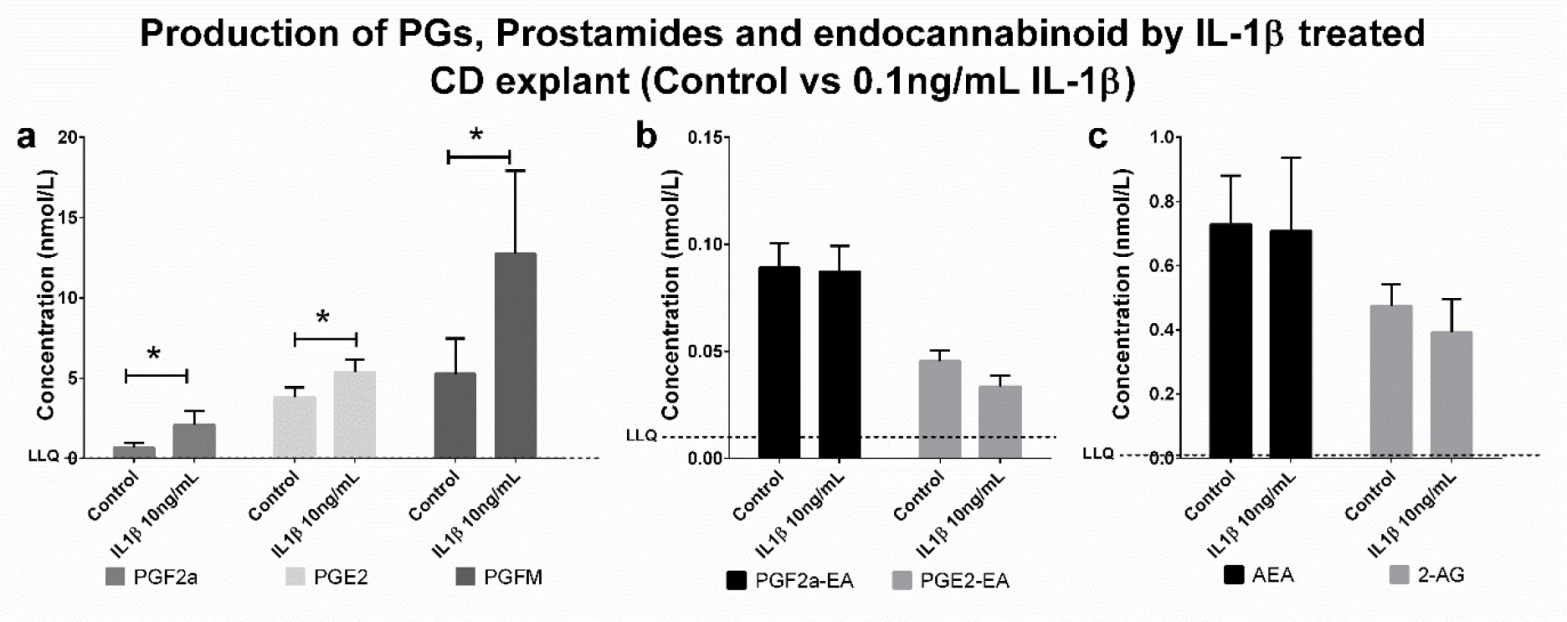
Production of PGs, Prostamides and Endocannabinoids by IL-1β treated CD explants (control vs 10ng/mL IL-1β). LC-MS/MS detection of prostaglandins (PGF_2α_, PGE_2_ and PGFM), (b) prostamides (PGF_2α_-EA and PGE_2_-EA) and (c) endocannabinoids (AEA and 2-AG) production by choriodecidual explants treated with 10ng/mL IL-1β. Dashed line represents the lower limit of quantitation (LLQ). Significant differences in production of the compounds were determined by Wilcoxon signed-rank test and significance was defined as P<0.05 (* on graphs). Data were presented as the mean ± S.E.M.

**Fig 6:**
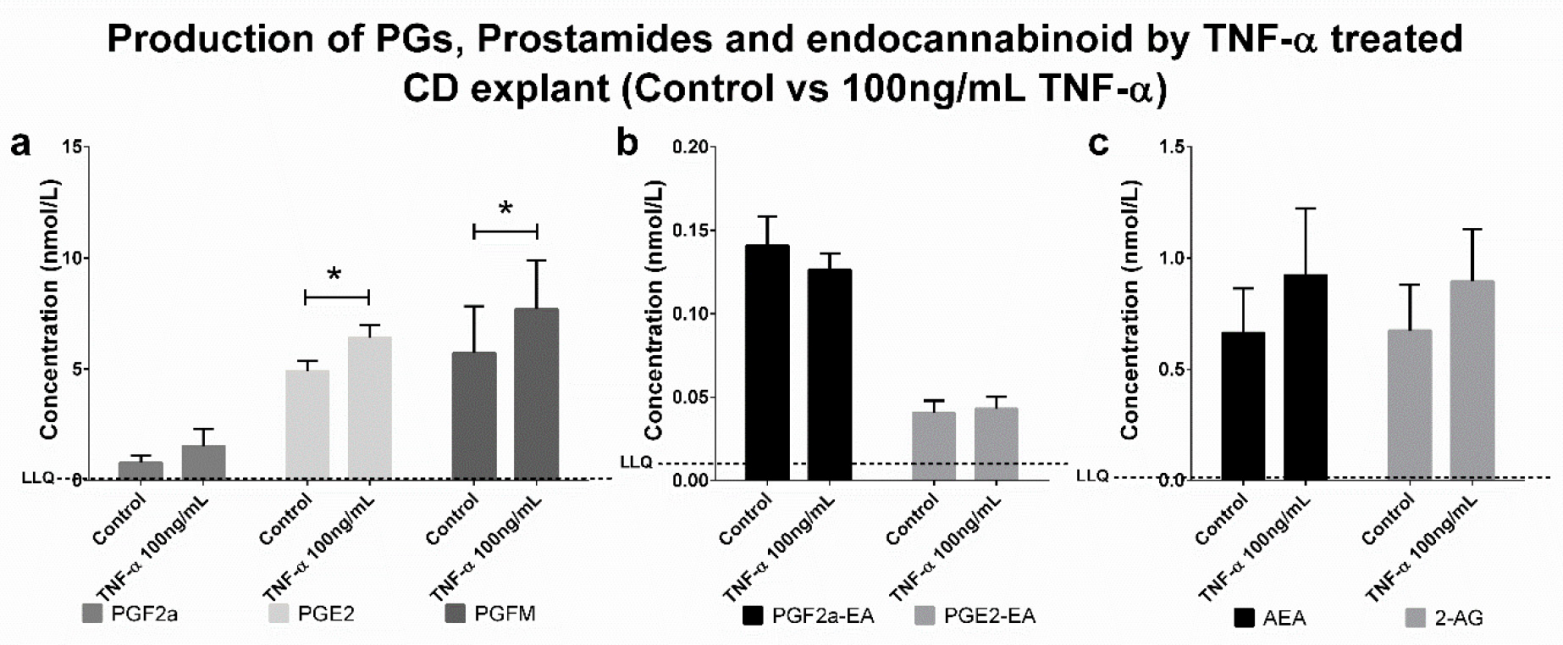
Production of PGs, Prostamides and Endocannabinoids by TNF-α treated CD explants (control vs 10ng/mL TNF-α). LC-MS/MS detection of prostaglandins (PGF_2α_, PGE_2_ and PGFM), (b) prostamides (PGF_2α_-EA and PGE_2_-EA) and (c) endocannabinoids (AEA and 2-AG) production by choriodecidual explants treated with 100ng/mL TNF-α. Dashed line represents the lower limit of quantitation (LLQ). Significant differences in production of the compounds were determined by Wilcoxon signed-rank test and significance was defined as P<0.05 (* on graphs). Data were presented as the mean ± S.E.M.

**Fig 7:**
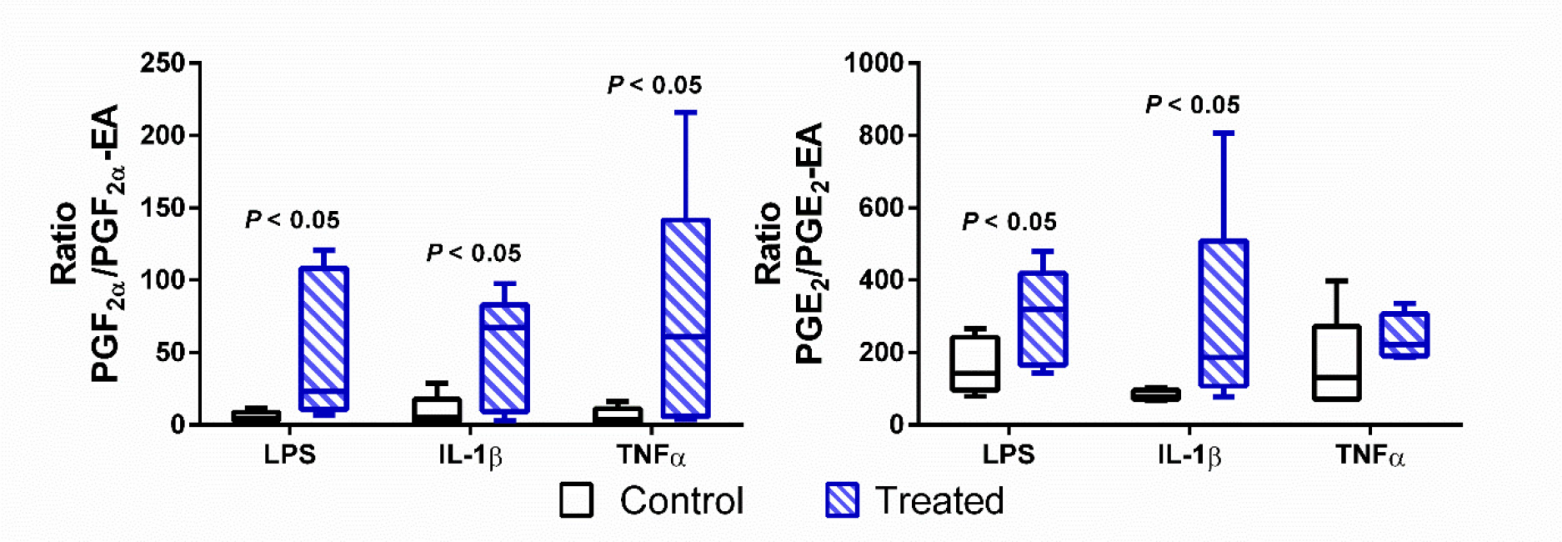
Ratio of Prostaglandin to prostaglandin ethanolamide production by LPS, IL-1β, or TNF-α treated choriodecidual explants. Production of eicosanoids (mean ratio of PGE_2_ and PGF_2α_ to ethanolamide metabolites, 25% and 75% quantiles with 95% confidence intervals, n = 5) by term choriodecidua after treatment with LPS, IL-1β, or TNF-α (in Blue; control in black). Data were analyzed using Wilcoxon signed-ranks tests.

Data were collected using positive/negative periods, which enabled the measurement of both prostaglandins (negative ions) and endocannabinoids and prostamides (positive ions) concomitantly (Fig 2). This approach is flexible for including additional lipid metabolites of interest in the future. A gradient elution under reverse-phase (C18) high-performance liquid chromatography conditions led to separation of individual peaks in under 7.5 min, which were then quantified using a matrix-matched standard curve (20–5,000 pmol/L). Mobile phase conditions were obtained from the literature [14, 15]. High specificity (i.e., unique mass pairs) and sensitivity (< 8 pg/mL for prostaglandins and <2 pg/mL for prostamides; see lower limit of quantification (LLOQ) in Table 1) enabled us to measure physiologically relevant levels of lipids in choriodecidual explants (Fig 3). Analysis of each lipid type is achieved by monitoring characteristic mass fragment pairs for each molecule at their distinct retention times (Rt; scheduled multiple reaction monitoring (MRM) transitions in Table 1). The concentrations of eicosanoids generated by chorio-decidual explants (in explant culture media) measured by LC-MS/MS data for explants treated with LPS, IL-1β and TNF-α are shown in Figs 4–6. As would be predicted all three treatmens stimulated the production of PGF_2α_, PGE_2_ and PGFM, with minor variations (Figs 4–6A). However, none of the inflammatory stimuli caused a significant change in the productions of prostamides or endocannabinoids (Figs 4–6B and 6C). Concentrations of prostaglandins, prostamides and endocannabinoids were well above the limit of detection of the assay (as indicated by the dashed line in Figs 4–6). The mean ratio of PGE_2_ and PGF_2α_ to ethanolamide metabolites produced by term choriodecidua after treatment with LPS, IL-1β, or TNF-α is given in Fig 7. Significant changes in the ratios were noted with all the treatments. These data suggest a higher proportion of PGE_2_ and PGF_2α_ are produced following treatment with LPS, IL-1β, or TNF-α.

**Table 1:** Optimized scheduled MRM pairs and parameters for eicosanoids.

	Rt (min)	Period 1 (Positive)		Period 2 (Negative)		Period 3 (Positive)			LLOQ (pg/mL)
**Endogenous lipids:**		Q1	Q3	Q1	Q3	Q1	Q3	CE	
**PGF**_**2α**_**-EA**	3.12	380.4	62.0					45	2
**PGE**_**2**_**-EA**	3.14	382.4	62.0					45	2
**PGF**_**2α**_	3.63			353.2	309.0			-28	8
**PGE**_**2**_	3.71			351.1	271.1			-24	8
**PGFM**	4.00			353.2	113.0			-36	8
**AEA**	5.11					348.4	62.0	25	1
**2-AG**	5.36					379.1	287.2	21	10
**Internal standards:**		Q1	Q3	Q1	Q3	Q1	Q3	CE	
**PGF**_**2α**_**-EA-*d4***	3.11	384.4	62.0					45	2
**PGE**_**2**_**-EA-*d4***	3.13	382.4	66.0					45	2
**PGF**_**2α**_**-*d4***	3.63			357.2	313.0			-28	8
**PGE**_**2**_**-*d4***	3.70			355.1	319.0			-24	8
**PGFM-*d4***	3.99			357.2	186.9			-36	8
**AEA-*d4***	5.11					352.4	66.0	25	1
**2-AG-*d8***	5.36					387.2	295.0	21	10

## Discussion

Our results are consistent with earlier work [12] and our hypothesis. We have now shown, with specific identification of eicosanoid products, that there is differential regulation of prostaglandin and prostamide production highlighted by a massive stimulation of prostaglandin E_2_, F_2α_ and FM production by IL-1β with no response in prostamide production (Fig 5). This is consistent with and provides further evidence for our hypothesis and is identical to the data in [12]. The significant enhanced differential response of prostamide production to IL-1b in the earlier paper by Glass et al [12] was only when added anandamide substrate was added to mimic a site of inflammation at which cells had been recruited that provided such substrate (as described below). Unfortunately, we have not replicated that part of the earlier study but aim to do so in the near future.

We provide unequivocal evidence that there is differential regulation of prostaglandin and prostamide biosynthesis in human choriodecidua in response to inflammatory stimuli. Each of the inflammatory mediators tested consistently provoked a preferential drive through the prostaglandin biosynthetic pathways. This is consistent with findings in amnion and an amnion derived cell line using crude chromatographic separation techniques [12]. It is interesting and valuable information since differences have been discerned in responses of fetal versus maternal tissues in cannabinoid stimulation of prostaglandin biosynthetic pathways [13]. Our data demonstrate that both amnion and choriodecidua respond similarly with this differential drive through the prostaglandin biosynthetic pathways. Moreover, and importantly, this has been shown using the “gold standard” of measurement by mass spectrometric means. How might this relate to the situation in vivo? These findings create doubt about the interpretation of data on prostaglandin biosynthesis in intrauterine tissues from pregnant women especially in the presence of an infection. The possibility is raised that separation of these products might lead to potential uses for their measurement in the diagnosis of preterm labor. The major identifiable cause of preterm labor is intrauterine infection [10], which may induce the secretion of cross-reacting prostamides, as anandamide release has been observed in response to hemorrhagic shock [16], LPS treatment of macrophages [17], and LPS challenge of human peripheral lymphocytes [18]. Likewise, COX-2 is induced by several inflammatory stimuli, such as IL-1β and LPS. These findings suggest that at the site of inflammation or infection, increases in both anandamide and COX-2 may synergistically induce secretion of PGE_2_-ethanolamide. Increased intrauterine prostaglandin production is considered a critical step in the process of human parturition. Hence it is vital to ascertain the results of experiments similar to those presented here, but determining the influence of additional amounts of anandamide and 2-AG on the rates of production and ratio of all end products.

Endocannabinoid signaling is tightly regulated throughout normal pregnancy. Plasma anandamide concentrations, as measured by MS, are markedly elevated in association with term labor [19, 20]. Interestingly, plasma anandamide concentrations measured in the first trimester of women presenting with signs of potential miscarriage are 3-fold higher in those who subsequently miscarry compared with those who progress to term [21]. This suggests that high plasma anandamide concentrations may lead to uterine activation and labor onset. Increased anandamide availability could be due to upregulated synthesis by N-acyltransferase and phospholipase D or reduced hydrolysis by fatty acid amide hydrolase (FAAH). In fact, FAAH protein and mRNA expression as well as activity in peripheral lymphocytes is reduced in women who miscarry spontaneously or fail to maintain pregnancy post-*in vitro* fertilization in the first trimester compared with gestational age-matched women undergoing voluntary pregnancy termination [22, 23].

Overall, however, the potential of prostaglandins, prostamides, and endocannabinoids to serve as clinically useful diagnostic and predictive factors for preterm labor may have been overlooked due to erroneous interpretation of immunoassay-related data. Examination of the similar molecular structures of key substances [24] demonstrates why immunoassays may have difficulty in distinguishing among these moieties.

## Conclusions

Our results cannot be applied to all physiological and pathophysiological situations but may certainly completely alter our thinking in others. The key factors will be the availability of substrate for the action of key enzymes of arachidonic acid metabolism–particularly recruitment of endocannabinoids via infiltrating cells as we mention earlier. Under these conditions and dependent upon the enzymes present then major products might be prostaglandins or prostamides or indeed other related substances e.g. from the lipoxygenase pathway. In a situation in which prostaglandins were being assessed for utility as diagnostics for e.g. preterm labor then the additional prostamides would register in assays but have little bioactivity on the myometrium and thus provide variability in results that would make the difference between clinical utility and not. This is especially true for pregnancy and labor and high risk pregnancies since the use of prostaglandins and inhibitors is widespread in clinical practice and it has always been a source of frustration that to date measurements were not useful diagnostically or prognostically. In future the measurement of products of the different pathways must be assessed using mass spectrometry as this is not compromised by antibody specificity or lack thereof. This may at least provide confirmation of identity and validation of results and although this is the “gold standard” and should be in regular use, that is not always possible and it should at least be done in a comparison and validation experiment initially. It remains possible that many of our ideas about prostaglandins and their roles physiological and pathophysiological mechanisms may be radically altered by this knowledge.
